# A commensal protozoan exacerbates acetaminophen-induced liver injury by producing free sphingosine

**DOI:** 10.1080/19490976.2026.2681811

**Published:** 2026-06-02

**Authors:** Xiaoqing Yan, Yusi Shen, Qian Sun, Xiuwen Zhu, Qiuchong Chen, Longxiang Liao, Yuan Zhou, Wanpeng Cheng, Zhen Shi, Zhuanzhuan Liu, Yanxia Wei, Xiangye Liu, Yugang Wang, Yanbo Kou

**Affiliations:** a Laboratory of Emergency Medicine, Second Clinical Medical College, Xuzhou Medical University, Xuzhou, China; b Department of Emergency Medicine, The Affiliated Hospital of Xuzhou Medical University, Xuzhou, China; c Jiangsu Key Laboratory of Immunity and Metabolism, Jiangsu International Laboratory of Immunity and Metabolism, Department of Pathogenic Biology and Immunology, School of Basic Medical Sciences, Xuzhou Medical University, Xuzhou, China; d Department of General Practice, The Affiliated Xuzhou Municipal Hospital of Xuzhou Medical University, Xuzhou, China; e Xuzhou Key Laboratory of Laboratory Diagnostics, Medical Technology School, Xuzhou Medical University, Xuzhou, China

**Keywords:** Gut microbiota, commensal protists, sphingosine, gut–liver axis, liver injury

## Abstract

The mechanisms by which the gut microbiota influence host disease outcomes remain poorly understood. As an integral yet overlooked component of this microbial community, the role of gut commensal protists in host physiology and pathology is even more ambiguous. Here, we show that the protozoan *Tritrichomonas musculis* (*T. mu*) remotely increases host sensitivity to drug-induced liver injury via the production of free sphingosine. Inhibiting sphingosine kinases (SPHKs) with PF543 or K145, or antagonizing sphingosine 1-phosphate receptor (S1PR) with FTY720, abolished the effect of *T. mu*-mediated exacerbation of acetaminophen (APAP)-induced liver injury (AILI), pointing to a sphingosine-SPHK1/2-S1P-S1PR axis underlying *T. mu'*s remote modulation of hepatic drug sensitivity. Moreover, using U-¹³C‑palmitic acid and U-¹³C‑glucose metabolic flux tracing, we propose a novel model for sphingosine synthesis in *T. mu*. In this model, *T. mu* synthesizes sphingosine by using oxaloacetate, likely generated via the glyoxylate cycle, as a two‑carbon unit donor, rather than directly employing intact palmitic acid to supply the long‑chain carbon skeleton. Collectively, these findings not only deepen our understanding of how the gut microbiota, particularly the underappreciated gut protists, influence extra-intestinal disease outcomes via the gut-distal organ axis, but also reveal the tip of the iceberg regarding the unique metabolic pathways of gut commensal protists.

## Graphical abstract

**Figure:**
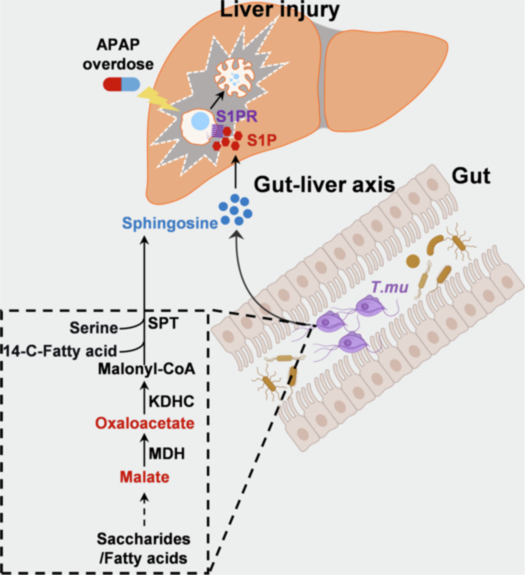

## Introduction

The gut microbiota is a complex microbial ecosystem that exerts broad influences on the host's intestinal tract and the distant organs.^1^ Beyond the well-characterized bacterial community, the gut microbiota also includes other communities, particularly the overlooked protists. With the increasing understanding of the integrity of the gut microbiota, the role of gut commensal protists is also coming to light. Colonization of commensal protists exerts profound impacts on the host, including remodeling the small intestinal architecture, defending against pathogenic infections, regulating host glucose homeostasis, and reshaping the host immune landscape.^2‐9^ These accumulating evidences reveal that the neglected commensal protists, much like bacteria, modulate host responses as well.

Overdose of acetaminophen (APAP), a widely used antipyretic and analgesic drug, is a leading cause of drug-induced liver injury and can result in severe hepatotoxicity. Individual susceptibility to APAP-induced liver injury (AILI) is closely tied to variations in the gut microbiome.^10‐13^

Here, we define a critical role for the commensal protozoan *T. mu* in exacerbating drug-induced liver injury. This exacerbation is attributed to *T. mu*'s ability to alter the host intestinal metabolome, notably by elevating sphingosine levels. Supplementation of sphingosine in vitro aggravates APAP-induced hepatocyte death. Consistently, both inhibiting the biotransformation of sphingosine to sphingosine-1-phosphate (S1P) and antagonizing S1P receptor (S1PR) were shown to attenuate *T. mu*‑mediated exacerbation of host liver injury. Furthermore, we discovered that *T. mu* can synthesize sphingosine, apparently utilizing two‑carbon units derived from saccharides or long‑chain fatty acids through a previously unreported pathway. These findings thus not only extend our knowledge of how an underappreciated commensal protozoan mediates drug-induced extra-intestinal tissue injury, but also advance our understanding of the unique metabolic pathways within the overlooked gut microbial community.

## Materials and methods

### Animal studies

All the animal studies were approved by the Institutional Animal Care and Treatment Committee of Xuzhou Medical University (SCXK (Su) 2020−0048). Mice were housed under controlled conditions (22° C ± 2 °C, 40%–70% humidity, 12-h light/dark cycle) with free access to food and water. Male C57BL/6 mice (6 to 8 weeks old) were purchased from Vital River Laboratory Animal Technology.

For the AILI, APAP (HY-66005, MCE) was intraperitoneally administered at a dose of 500 mg/kg (for acute liver injury) or 700 mg/kg (lethal dose).

To deplete gram-negative and -positive bacteria, 200 mg/L nalidixic acid (A610363, Sangon Biotech) and 500 mg/L vancomycin (A600983, Sangon Biotech) were added to the drinking water accompanied by daily colistin sulfate (C805491, Macklin) gavage (600 μg/mouse). To deplete the colonized *T. mu*, 3 g/L metronidazole (A600633, Sangon Biotech) was added to the drinking water.

For in vivo suppression of S1PR and SPHK1, 10 mg/kg/d FTY720 hydrochloride or 10 mg/kg PF-543 citrate (HY-12005, HY-15425, MCE) were injected intraperitoneally 0.5 h before APAP challenge respectively. To suppress SPHK2, 50 mg/kg K145 hydrochloride (HY-15779A, MCE) was orally administrated 0.5 h before APAP challenge. For in vivo suppression of choline TMA lyase, mice were fed in the drinking water with 1% (vol/vol) 3,3-dimethyl-1-butanol (DMB; 183105, Merck) for 7 d before APAP challenge.

### *T. mu* quantification, isolation, and *in vivo* transfer

The quantification, isolation, purification, and *in vivo* transfer of *T. mu* were performed according to our previously established method.^6^ For the ^13^C-based metabolic flux analysis, an optimized medium containing a cocktail of broad-spectrum antibiotics (Table S1) was used to further purify and culture the protist.

### Wright–Giemsa staining

Samples were stained using the Wright–Giemsa staining kit (D010, Nanjing Jiancheng Bioengineering Institute).

### Serum biochemistry

The serum alanine aminotransferase (ALT) and aspartate aminotransferase (AST) were determined by commercially available diagnostic kits (A009, A010, Nanjing Jiancheng Bioengineering Institute).

### Histological examinations

Sections from formalin-fixed and paraffin-embedded liver tissues were stained with a Hematoxylin and Eosin (HE) Staining Kit (C0105S, Beyotime).

### Untargeted metabolomics analysis

Untargeted metabolomics analysis was conducted by Shanghai Applied Protein Technology. Cecal content was homogenized in ultrapure water before methanol/acetonitrile (1:1, v/v) addition. After sonication, the samples were incubated at −20 °C. Then the samples were centrifuged for protein precipitation, and the resulting supernatants were collected and freeze-dried for subsequent mass spectrometry analysis. All prepared samples were separated by ultra-high-performance liquid chromatography (UHPLC) using an Agilent 1290 Infinity LC system (Agilent Technologies). The eluates were analyzed on a Triple TOF 5600 mass spectrometer (AB SCIEX). Detection was performed using electrospray ionization (ESI) in both positive and negative ion modes.

## ^13^C-labeled untargeted metabolic flux tracing analysis

For ^13^C-labeled untargeted metabolic flux tracing analysis, 5 g/L [U-^13^C_6_] glucose or 200 μM [U-^13^C_16_] palmitic acid (S25147, T80363, Shanghai Yuanye Bio-Technology) were supplemented to the optimized medium with equivalent unlabeled glucose or palmitic acid as control. After a 48 h-culture, the *T. mu* cells were harvested for the metabolic flux analysis.

The metabolic flux tracing analysis was performed by Shanghai Biotree biomedical technology. Briefly, the *T. mu* cells were placed on dry ice and fast quenched with −80 °C pre-cooled extraction solution [methanol/acetonitrile/water (2:2:1, v/v)]. After being incubated at −40 °C, the samples were vortexed followed by centrifugation at 4 °C. The supernatants were collected and evaporated. The dried extracts were reconstituted in acetonitrile/water (1:1, v/v) solution, followed by sonication and centrifugation to remove insoluble materials. LC-MS/MS analysis was performed on a Thermo Vanquish UHPLC system.

### Quantitation of sphingosine and S1P

Serum, cecal content, and liver tissue (homogenized) samples were subjected to two rounds of liquid-liquid extraction using methyl tert-butyl ether/methanol (5:1, v/v). The combined supernatants were lyophilized under vacuum, and the dried residues were reconstituted in methanol. After centrifugation at 13,000 × *g* for 10 min at 4 °C, the supernatant was collected for subsequent UPLC-MS/MS analysis. UPLC-MS/MS analysis was performed using an AB Sciex ExionLC liquid chromatography system coupled with a Triple Quad 3500 mass spectrometer. For sphingosine detection, mobile phase A consisted of acetonitrile/water (6:4, v/v) containing 10 mM ammonium formate, and mobile phase B consisted of acetonitrile/isopropanol (1:9, v/v). For sphingosine-1-phosphate (S1P) detection, mobile phase A was water with 0.1% formic acid, and mobile phase B was methanol with 0.1% formic acid.

### Bacterial microbiome analysis

16S rDNA sequencing was performed by Shanghai Majorbio Bio-Pharm Technology. The 16 S bacterial rRNA genes were amplified using 338 F (5′-ACTCCTACGGGAGGCAGCAG-3′) and 806R (5′-GGACTACHVGGGTWTCTAAT-3′). Sequencing libraries were generated through the NEXTFLEX Rapid DNA-Seq Kit (Bioo Scientific). The library was sequenced on an Miseq PE300/NovaSeq PE250 platform (Majorbio). Sequencing reads were clustered into OTUs at 97% identity and then taxonomically classified with the RDP classifier against the Silva138.1/16s_bacteria database at a 70% confidence threshold. Further analysis was carried out using the Majorbio Cloud platform.

### Cell culture and treatment

AML12 cell line was kindly provided by Dr. Chao Yan (Xuzhou Medical University). Cells were cultured in DMEM supplemented with 5 μg/mL insulin, 40 ng/mL dexamethasone, 10% FBS (Gibco, 16000-044), and 1% penicillin/streptomycin. For cell death and viability assays, cells were treated with sphingosine (HY-101047, MCE) or TMAO (317594, Merck) together with 5 mM APAP for 48 h followed by LDH and CCK-8 assays through the commercial lactate dehydrogenase assay kit (A020-2-2, Nanjing Jiancheng Bioengineering Institute) and Cell Counting Kit-8 (BS350B, Biosharp). For flow cytometric live/dead staining, cells were treated with APAP plus sphingosine or TMAO for 24 h, and stained using the Zombie Aqua™ Fixable Viability Kit (423101, Biolegend). For western blot assay, cells were treated with APAP plus sphingosine or TMAO for 12 h, and the antibodies used were as follows: anti-β-Actin, PERK, p-PERK, JNK, p-JNK, ASK1, p-ASK1 (4967/3192/3179/9252/4668/3762/3765, Cell signaling technology) and Anti-CHOP (WL00880, Wanleibio).

### Statistical analysis

Data were presented as mean ± SEM and analyzed using Prism (GraphPad Software, La Jolla, CA, USA). When the assumptions of normality and homogeneity of variance were violated, non-parametric tests were employed instead. Statistical significance was established at *p* < 0.05.

## Results

### *T .mu* is capable of exacerbating APAP-induced liver injury of host

**Figure 1.**
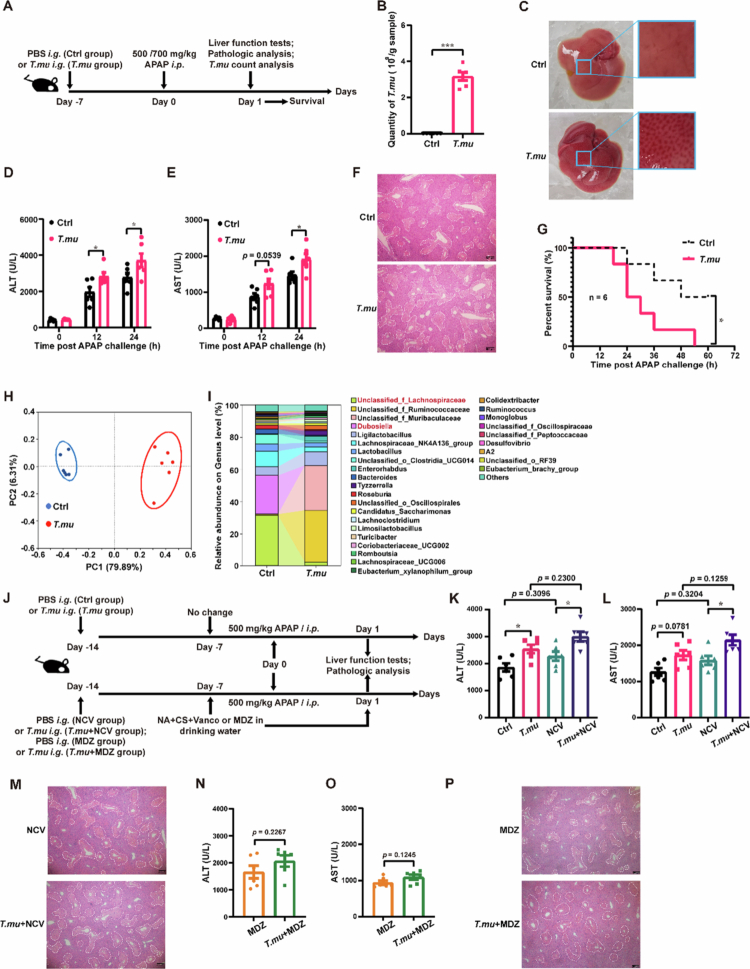
Colonization with *T. mu* exacerbates the severity of host AILI. (a) Schematic of the experimental setup for (b–g). (b) The number of *T. mu* in the cecal content. (c) Macroscopic appearance of the liver. (d and e) Serum levels of ALT (d), and AST (e) before and after 500 mg/kg APAP challenge. (f) Representative images of HE-stained liver sections from mice after APAP challenge, scale bars = 100 mm. (g) Survival after lethal dose of APAP challenge. (h) Principal coordinates analysis conducted on genus levels. (i) The average relative abundance on genus levels. (j) Schematic of the experimental setup for (k*–*p). NA nalidixic acid, CS colistin sulfate, Vanco vancomycin, MDZ metronidazole. (k and l) Serum levels of ALT (k), and AST (l) 24 h after APAP challenge, NCV NA + CS + Vanco. (m) Representative images of HE-stained liver sections after APAP challenge. (n and o) Serum levels of ALT (n), and AST (o) after APAP challenge. (p) Representative images of HE-stained liver sections after APAP challenge. All data are shown as mean ± SEM, *n* = 6. Mann–Whitney test (b), Two-way ANOVA (d and e), Log-rank (Mantel–Cox) test (g), One-way ANOVA (k and l) or Two-sided Student's *t*-test (n and o) were performed. *p < 0.05, **p < 0.01, ***p < 0.001.

We previously demonstrated the presence of *T. mu* in the GI tract of our in-house bred C57BL/6 (B6) mouse.^6^ ^,^ ^8^ ^,^ ^14^ Compared to *T. mu*-free B6 mice obtained from Vital River Laboratories (VRL, Beijing, China), our in-house bred mice demonstrated heightened susceptibility to APAP hepatotoxicity (Figure S1). Considering the influence of gut microbiota on drug-induced liver injury, and *T. mu* colonization results in a notable remodeling of the host intestinal microbiota,^6,10,12‐15^ the potential association between *T. mu* and APAP-induced liver injury (AILI) was examined. After adoptive transfer of purified *T. mu* cells, mice were challenged with APAP (Figure 1A and B). The *T. mu*-colonized mice showed exacerbated liver injury after APAP challenge as evidenced by serum liver function tests and histopathological analysis (Figure 1C–F). Consistently, *T. mu* colonization compromised survival following a lethal APAP challenge (Figure 1G).

While *T. mu* colonization significantly remodeled the gut bacteriome (Figure 1H and I), including reducing potentially protective taxa like *Lachnospiraceae* and increasing detrimental taxa like *Dubosiella,* ^16^ ^,^ ^17^ antibiotic depletion of Gram-positive and -negative bacteria did not mitigate the *T. mu*-mediated exacerbation of AILI (Figure 1J–M). Accordingly, the exacerbated liver injury phenotype disappeared following *T. mu* clearance with metronidazole treatment (Figure 1J and N–P, Figure S2). Collectively, these findings suggest that despite altering the gut bacterial community, *T. mu* itself is adequate to exacerbate AILI.

### *T. mu* drives accumulation of free sphingosine and choline in the host gut

**Figure 2.**
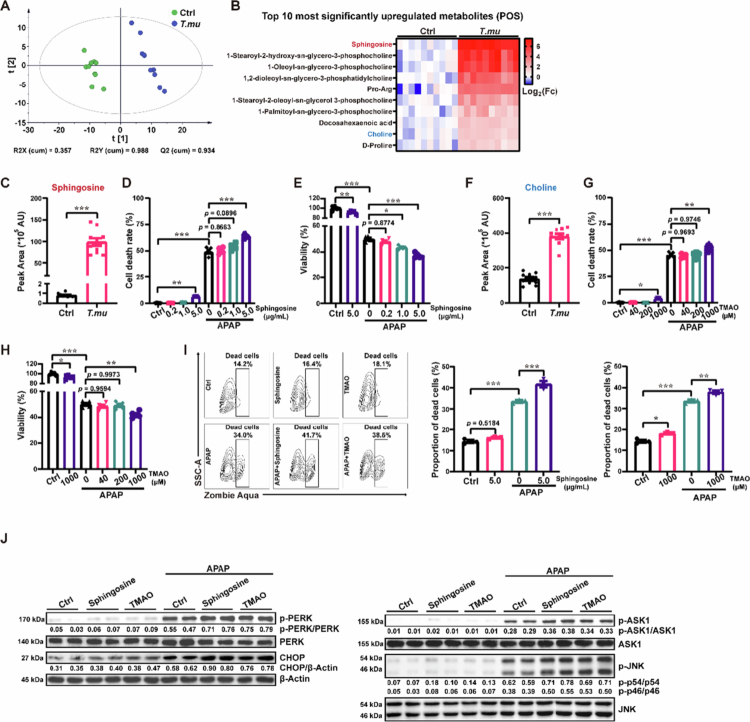
*T. mu* colonization raises intestinal sphingosine and choline. (a) Partial least squares discriminant analysis performed on the cecal content metabolite composition. (b) The top 10 upregulated metabolites in positive ion mode. (c) Free sphingosine levels in cecal contents. (d and e) Cell death rate (d) and viability (e) of AML12 cells after APAP challenge combined with a sphingosine concentration gradient. (f) Free choline levels in cecal contents. (g and h) AML12 Cell death (g) and viability (h) after APAP plus TMAO gradient co-treatment. (i) Analysis of the proportion of dead cells in AML12 cells treated with APAP and sphingosine or TMAO by flow cytometry-based live/dead staining. (j) Immunoblot analysis of PERK–CHOP and ASK1–JNK pathways activation in AML12 cells following co‑treatment with APAP and either sphingosine (5 μg/mL) or TMAO (1000 μM). All data are shown as mean ± SEM, *n* = 10 for A, B, C, F, *n* = 5 for D, E, G, H, and *n* = 3 for I. Mann–Whitney test (c), One-way ANOVA (d, e, g, h and i) or Two-sided Student's *t*-test (f) were performed. *p < 0.05, **p < 0.01, ***p < 0.001.

Beyond the microbiota themselves, gut metabolite composition critically modulates hepatic responses via the gut-liver axis. Untargeted metabolomics revealed that *T. mu* colonization significantly reshaped the intestinal metabolome (Figure 2A). Among altered metabolites, free sphingosine increased most markedly in positive ion mode (Figure 2B and C, and Figure S3). Prompted by this, we found that sphingosine dose-dependently exacerbated APAP-induced hepatocyte death in vitro (Figure 2D and E), identifying it as a potential mediator. Additionally, free choline was elevated as well (Figure 2B and F). Given our prior finding that *T. mu*-derived choline perturbs host metabolism via the choline-trimethylamine (TMA)-trimethylamine oxide (TMAO) axis,^8^ and that TMAO can activate injurious pathways,^18^ we tested TMAO, the derivative of choline, in vitro. A high concentration (1000 μM) of TMAO also aggravated APAP-induced hepatocyte death (Figure 2G and H), suggesting choline metabolism as another potential exacerbating pathway. Consistent with this, flow cytometry-based live/dead staining and the assessment of activation of the PERK-CHOP and ASK1-JNK signaling pathways (two key signaling cascades implicated in APAP-induced hepatocyte death) further corroborated that sphingosine and the choline-derived metabolite TMAO exacerbate APAP-induced hepatocyte injury (Figure 2I and J).

### *T. mu* exacerbates APAP-induced liver injury via the sphingosine-SPHK1/2-S1P-S1PR signaling axis

**Figure 3.**
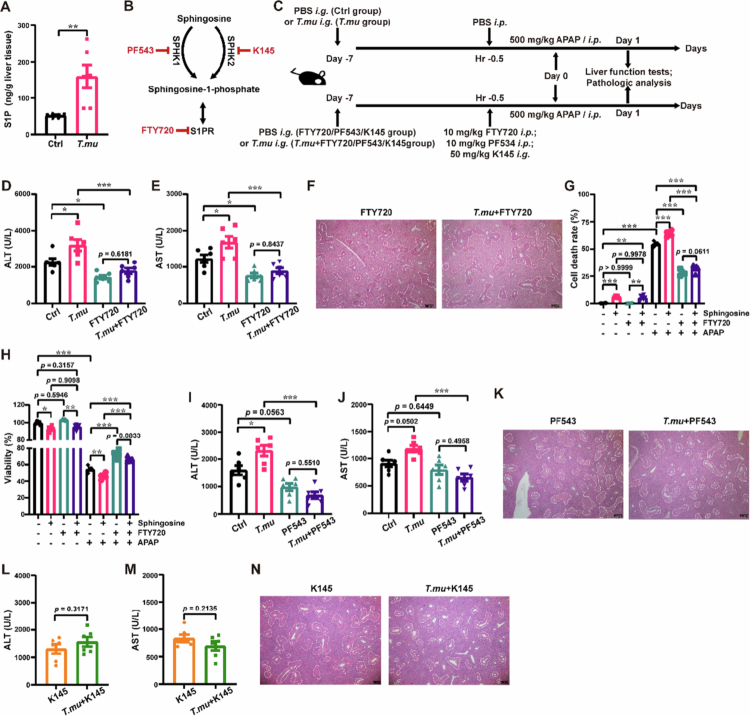
*T. mu* exacerbates AILI in a sphingosine-SPHK1/2-S1P-S1PR axis dependent manner. (a) The S1P levels in liver tissues were monitored by UPLC-MS/MS. (b) Schematic of the sphingosine metabolic pathways. (c) Schematic of the experimental setup. (d–f, and i–n) After *T. mu* colonization, 0.5 h prior to APAP challenge, mice received a single dose of vehicle, FTY720 (d–f), PF543 (i–k), or K145 (l–n). Serum ALT (d, i, l) and AST (e, j, m) levels were measured, and liver tissues were subjected to HE staining (f, k, n). (g and h) AML12 Cell death (g) and viability (h) before and after co-treatment with APAP plus 5 μg/mL sphingosine and 5 μM FTY720. All data are shown as mean ± SEM, *n* = 6. Mann–Whitney test (a), One-way ANOVA (d, e, and g–j) or Two-sided Student's *t*-test (l and n) were performed. *p < 0.05, **p < 0.01, ***p < 0.001.

To test whether elevated choline mediates *T. mu'*s exacerbation of AILI in vivo, we employed pathway-specific inhibitor to disrupt choline metabolism (Figure S4A and B). Although inhibition of the choline-TMA bioconversion slightly reduced AILI, it did not prevent *T. mu* from exacerbating AILI (Figure S4C and D), ruling out elevated choline as the primary mediator. We observed that colonization by *T. mu* led to a time‑dependent increase in sphingosine levels in the cecal contents and a corresponding rise in serum ALT/AST activities, as well as elevated sphingosine‑1‑phosphate (S1P) levels in liver tissues (Figure S5 and Figure 3A). Once S1P receptor (S1PR) was antagonized by FTY720, the exacerbating effect of *T. mu* on AILI was abolished (Figure 3B–F). Consistently, in vitro treatment with FTY720 counteracted the sphingosine-aggravated APAP-induced hepatocyte injury (Figure 3G and H). Furthermore, blocking the bioconversion of sphingosine into S1P by inhibiting Sphingosine kinase 1/2 (SPHK1/2) also nullified the exacerbating effect of *T. mu* on AILI as well (Figure 3B and C, I–N). Interestingly, SPHK1 and SPHK2 appear to contribute comparably to the process of *T. mu*-aggravated AILI, as inhibition of either SPHK1 or SPHK2 alone was sufficient to abolish the *T. mu*-mediated exacerbation of AILI (Figure 3I–N). These observations overall suggest that *T. mu* exacerbates AILI in a sphingosine-SPHK1/2-S1P-S1PR axis dependent manner.

### *T. mu* synthesizes free sphingosine

**Figure 4.**
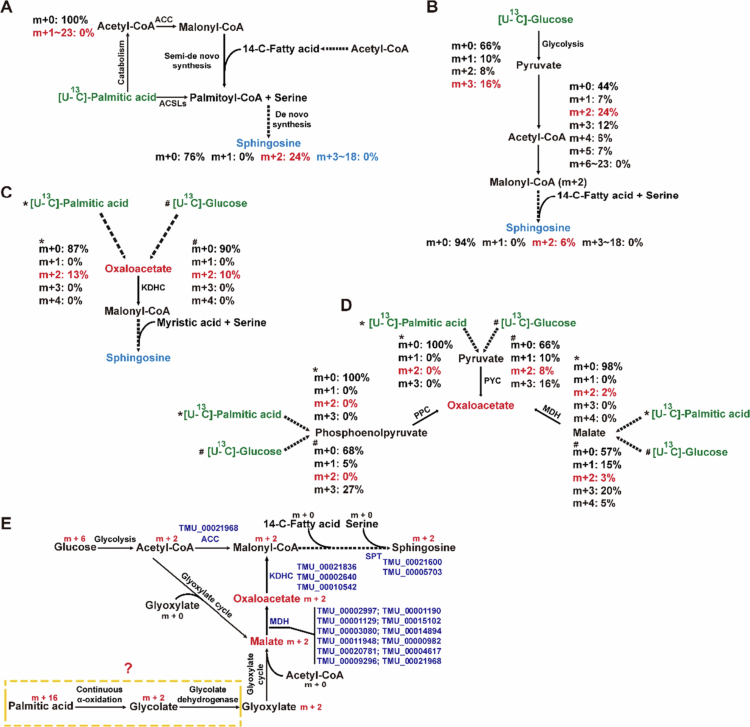
*T. mu* generates sphingosine through a novel mechanism. (a) Schematic diagram depicting the metabolic pathway from palmitic acid to sphingosine, traced using U-¹³C-palmitic acid (b) Schematic depicting the metabolic pathway from glucose to malonyl-CoA and its subsequent incorporation into sphingosine, traced using U-^13^C-glucose. (c) Two ^13^C-labeled-oxaloacetate was detected in both the U-^13^C-palmitate and U-^13^C-glucose metabolic flux systems. (d) Identifying potential sources of oxaloacetate via metabolic flux analysis. (e) The proposed sphingosine biosynthetic pathway in *T. mu*. * indicates the U-^13^C-palmitic acid metabolic flux system, and # indicates the U-^13^C-glucose metabolic flux system. Analysis was performed on pooled samples, (*n* = 3).

Sphingosine is synthesized de novo from Palmitoyl-CoA or obtained from dietary sphingolipids.^19^ ^,^ ^20^ To test if *T. mu* directly generates free sphingosine, we performed in vitro ^13^C-tracer analysis using U-^13^C-palmitic acid. Unexpectedly, only two-^13^C-labeled sphingosine was detected, rather than the 16-^13^C-labeled sphingosine from intact palmitic acid incorporation (Figure 4A). Furthermore, the absence of ^13^C-labeled acetyl-CoA (a key β-oxidation product) indicates that *T. mu* lacks a functional β-oxidation pathway (Figure 4A). Despite possessing alternative α- and ω-oxidation for fatty acid degradation (Figure S6), lacking of subsequent β-oxidation leaves the source of the ^13^C-labeled two‑carbon units unclear. These findings suggest that *T. mu* can synthesize sphingosine, likely through elongation of a 14‑carbon fatty acid using the two‑carbon units rather than via direct palmitic acid incorporation.

Since the two-carbon unit donors (e.g., acetyl-CoA-derived malonyl-CoA) required for fatty acid elongation can be produced from glucose catabolism (Figure 4B), we next employed U-^13^C-labeled glucose for metabolic flux tracing. *T. mu* possesses a complete glycolytic enzyme system (Figure S7). As hypothesized, *T. mu* produced ^13^C-labeled sphingosine, but as with the palmitic acid system, only the two-^13^C‑labeled form was detected (Figure 4B). The two-carbon unit incorporated into sphingosine is reasonably speculated to originate from glucose-derived two-^13^C-labeled acetyl-CoA via malonyl-CoA. Notably, while U-^13^C-palmitate produced no ^13^C-acetyl-CoA, two-^13^C-labeled sphingosine was detected (Figure 4A), suggesting acetyl-CoA is not the sole primordial two‑carbon donor. Malonyl‑CoA, the direct elongation unit, can also originate from oxaloacetate via the α-Keto acid dehydrogenase complex (KDHC).^21^ Since two-^13^C-oxaloacetate was detected in both tracer systems (Figure 4C), *T. mu* likely uses oxaloacetate as an alternative two‑carbon unit donor for sphingosine synthesis.

Oxaloacetate synthesis occurs via several routes: from pyruvate, phosphoenolpyruvate, or malate (Figure 4D). Three-^13^C-labeled pyruvate derived from glycolysis was not further carboxylated into three-^13^C-labeled oxaloacetate (Figure 4C and D). While ^13^C-labeled glucose produced two-^13^C-labeled pyruvate via some unknown metabolic pathway, no two-^13^C-labeled pyruvate was detected in the ^13^C-labeled palmitic acid system (Figure 4D). This rules out the pyruvate carboxylase (PYC) pathway. As an anaerobe, *T. mu* is expected to favor the phosphoenolpyruvate carboxylase (PPC) route, however, no two‑^13^C‑labeled phosphoenolpyruvate was detected (Figure 4D). In contrast, two‑^13^C‑labeled-malate was present in both tracer systems, indicating oxaloacetate is instead produced via the malate dehydrogenase (MDH) pathway. Given the incomplete TCA cycle in the anaerobic *T. mu*, the co‑detection of two‑^13^C‑aconitate and isocitrate suggests that malate may originate from the glyoxylate cycle (Figure S8). Since *T. mu* possesses an α-oxidation pathway, we propose that palmitic acid might be processed through continuous α-oxidation and glycolate dehydrogenation to form glyoxylate. Together with supporting genomic evidence (Table S2), our results indicate that *T. mu* may employ a relatively distinct pathway for sphingosine synthesis (Figure 4E).

## Discussion

Although gut microbiota profoundly impacts host homeostasis,^22‐24^ the role of its often-overlooked eukaryotic members, particularly protists, remains unclear.^2^ Thus, elucidating how symbiotic gut protists modulate host responses is essential for advancing our understanding of host-microbiota interactions. In this study, we demonstrate how the commensal protist in gut can modulate drug-induced extra-intestinal organ injury in the host. Our findings indicate that the protozoan *T. mu* synthesizes free sphingosine via a relatively distinct biosynthetic pathway. Colonization by *T. mu* elevates luminal free sphingosine levels, thereby exacerbating AILI through the sphingosine-SPHK1/2-S1P-S1PR axis.

While *T. mu* colonization alters the gut bacterial community, a factor linked to drug-induced liver injury,^6^ ^,^ ^14^ ^,^ ^25^ ^,^ ^26^ we found that depleting Gram-positive and -negative bacteria did not abolish its exacerbation of AILI, indicating that *T. mu* likely worsens APAP hepatotoxicity completely independent of gut bacteria. However, different gut bacterial backgrounds can lead to divergent outcomes, even with the same intervention in different laboratories.^27^ ^,^ ^28^ Therefore, the potential influence of bacterial community alterations on *T. mu*-exacerbated AILI cannot be entirely excluded, but current evidence indicates that *T. mu* itself is indeed capable of directly aggravating host AILI.

A common mechanism by which the gut microbiota affects host AILI is through the production of specific metabolites.^27‐29^ We show that *T. mu* colonization elevates intestinal sphingosine and exacerbates injury through the sphingosine-SPHK1/2-S1P-S1PR axis, as blockade of this axis abolished the phenotype. While SPHK1 is known to be involved,^30^ we found that not only SPHK1 but also SPHK2 play a critical role, since SPHK2 inhibition similarly prevented *T. mu*-mediated exacerbation.

Sphingosine synthesis in *T. mu* involves an unreported pathway. We found that two‑carbon units from both glucose and palmitic acid are incorporated, confirming *T. mu*’s autonomous synthesis capacity. Contrary to typical sphingosine synthesis, *T. mu* does not incorporate intact palmitic acid but only its two‑carbon units. Based on U‑^13^C‑palmitic acid tracing, we propose that *T. mu* likely lacks a functional β‑oxidation system. Instead, oxaloacetate generated via the glyoxylate cycle, rather than acetyl‑CoA from β‑oxidation, serves as the initial two‑carbon donor for sphingosine synthesis. This hypothesis was supported by U‑^13^C‑glucose tracing and is partly consistent with the genomic profile of *T. mu*. However, due to the absence of a feasible genetic manipulation system for *T. mu*, the above conclusions (particularly the proposed glyoxylate cycle-dependent pathway for malate production) are based primarily on metabolic flux analysis and lack direct enzymatic confirmation. Therefore, this hypothesis requires further experimental validation.

Taken together, we have proposed a relatively distinct sphingosine synthesis pathway in *T. mu*. Sphingosine produced by *T. mu* exacerbates host AILI through the sphingosine-SPHK1/2-S1P-S1PR axis. Our findings regarding how *T. mu* modulates drug-induced liver injury in the host carry broad implications, demonstrating that the intestinal microbiota, extending beyond bacterial communities, can initiate a gut-tissue axis to remotely regulate the sensitivity of distal organs to drug-induced injury. Furthermore, we propose a novel working model for sphingosine synthesis in *T. mu*, which advances our understanding of unique metabolic pathways in often-overlooked unicellular protozoa.

## Supplementary Material

Supplementary Material Supplementary Information_20260327.docx

## Data Availability

The 16S rDNA sequencing data has been deposited in the GenBank Sequence Read Archive database (www.ncbi.nlm.nih.gov/sra/?term=PRJNA1394237). The re-annotated genomic information is provided in Science Data Bank (www.scidb.cn/en/anonymous/WjdmYVFm). Any additional information required to reanalyze the data reported in this paper is available from the corresponding author, Yanbo Kou (fightingkyb@163.com).
