# Supplementary material for: A commensal protozoan exacerbates acetaminophen-induced liver injury by producing free sphingosine
Source: Gut Microbes. 2026 Jun 2;18(1):2681811. doi: 10.1080/19490976.2026.2681811 (PMC13240944; doi:10.1080/19490976.2026.2681811)
Supplement: Supplementary Material — Supplementary Information_20260327.docx [file KGMI_A_2681811_SM7277.docx]

**Supplementary Information**

**A commensal protozoan** **exacerbates acetaminophen-induced liver injury by producing free** **sphingosine.**

Xiaoqing Yan, Yusi Shen, Qian Sun, Xiuwen Zhu, Qiuchong Chen, Longxiang Liao, Yuan Zhou, Wanpeng Cheng, Zhen Shi, Zhuanzhuan Liu, Yanxia Wei, Xiangye Liu, Yugang Wang, Yanbo Kou


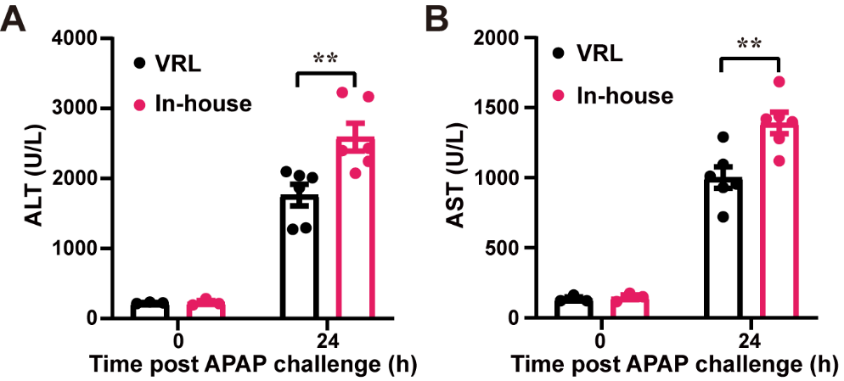


**Fig. S1 Liver injury induced by APAP were aggravated in the in-house reared B6 mice.** Serum levels of ALT **(A)**, and AST **(B)** in the *T.mu*-free B6 mice purchased from Vital River Laboratories (VRL) and our in-house reared sex- and age-matched B6 mice, n = 6. All data are shown as mean ± SEM. Two-way ANOVA was performed. * *p* < 0.05, ** *p* < 0.01.


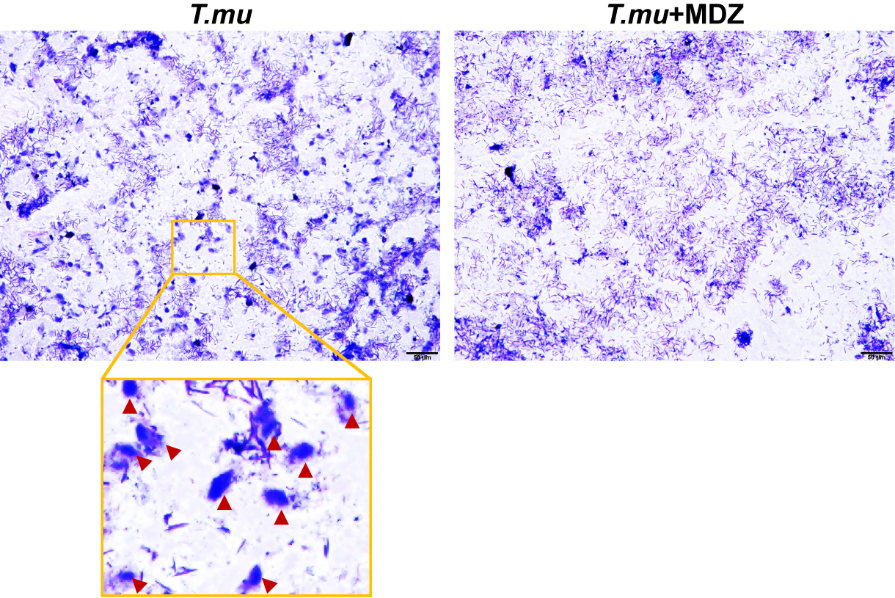


**Fig. S2 The colonized-*T.mu* was depleted via administration of metronidazole.** Wright-Giemsa staining of the cecal content from the *T.mu*-colonized mice and the metronidazole (MDZ) treated *T.mu*-colonized mice, scale bar = 100 μm. Inset graph shows the presence of *T.mu* protists. The red arrow heads indicate the stained *T.mu* protists.


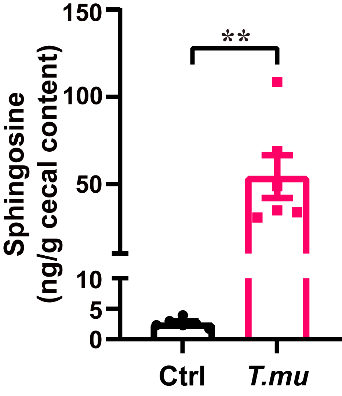


**Fig. S3 Determination of sphingosine content in cecal contents by UPLC-MS/MS.** UPLC-MS/MS quantification of sphingosine in cecal contents of *T.mu*‑colonized and control *T.mu*-free mice, n = 6. The data are shown as mean ± SEM. Two-sided Student’s t-test was performed. * *p* < 0.05, ** *p* < 0.01.


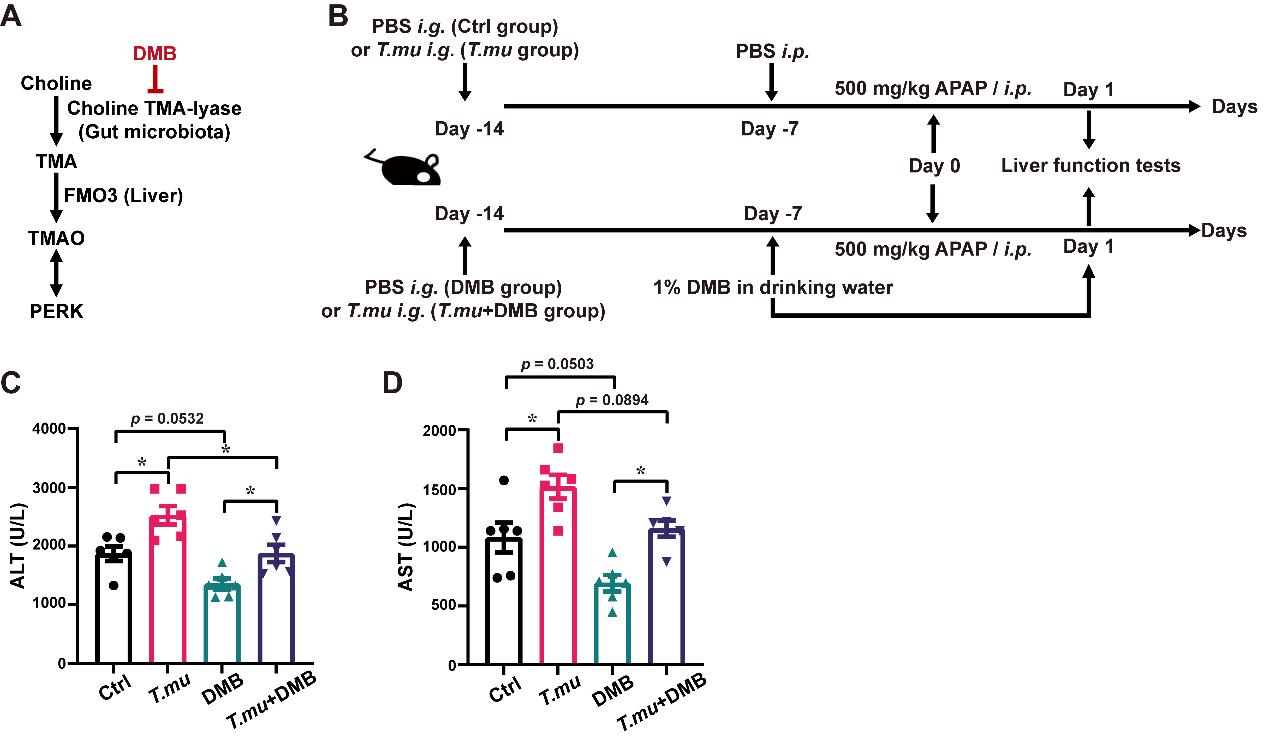


**Fig. S4 Inhibiting choline-TMA biotransformation did not abolish the *T.mu*-mediated exacerbation of APAP-induced liver injury.** **(A)** Schematic of the choline metabolic pathways. **(B)** Schematic of the experimental setup for *T.mu* colonization, 3,3-Dimethyl-1-butanol (DMB) treatment, and APAP challenge. **(C and D)** Serum levels of ALT (C), and AST (D) in the following groups 24 h after a 500 mg/kg APAP challenge: Ctrl mice, *T.mu*-colonized mice, DMB treating mice, and the *T.mu*-colonized DMB-treated mice, n = 6. All data are shown as mean ± SEM. One-way ANOVA with Tukey's post hoc test was performed. * *p* < 0.05.


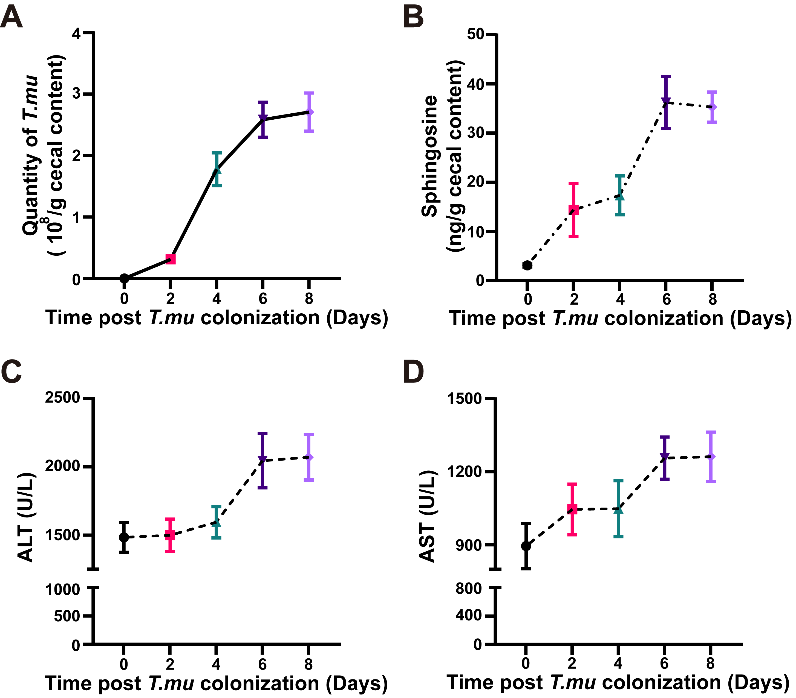


**Fig. S5 APAP-induced liver injury is aggravated with increasing *T.mu* colonization.** After *T.mu* colonization for different time periods (0, 1, 3, 5, and 7 days), mice were intraperitoneally injected with APAP at a dose of 500 mg/kg. Cecal contents were collected 24 hours later for *T.mu* counting **(A)** and sphingosine content determination **(B)**, and serum was collected for measurement of ALT **(C)** and AST **(D)** activities, n = 3. All data are shown as mean ± SEM.


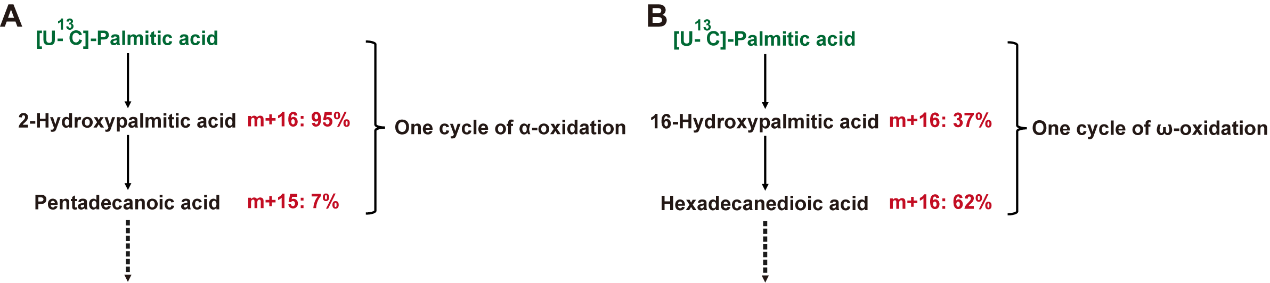


**Fig. S6 *T.mu* possesses both** **α- and ω-oxidation pathways for long-chain fatty acids.** Schematic diagram depicting the α- (A) and ω-oxidation (B) pathways of palmitic acid, as traced using U-¹³C-palmitic acid.


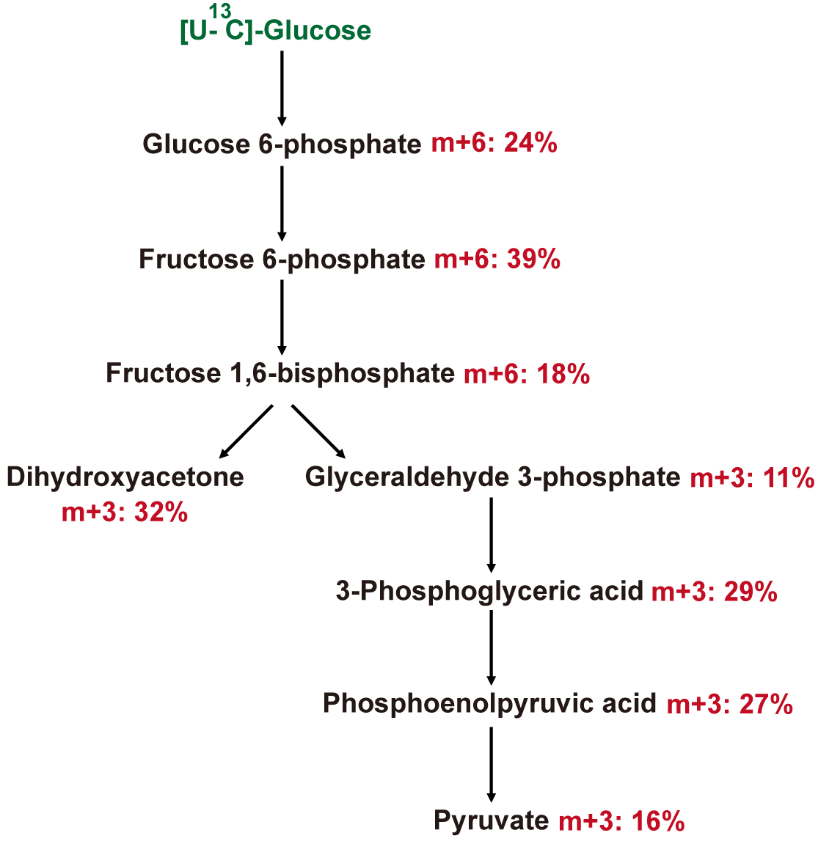


**Fig. S7 A complete glycolytic pathway is present in *T.mu*.** Schematic diagram depicting the glycolytic pathway from glucose, traced using U-¹³C-glucose.


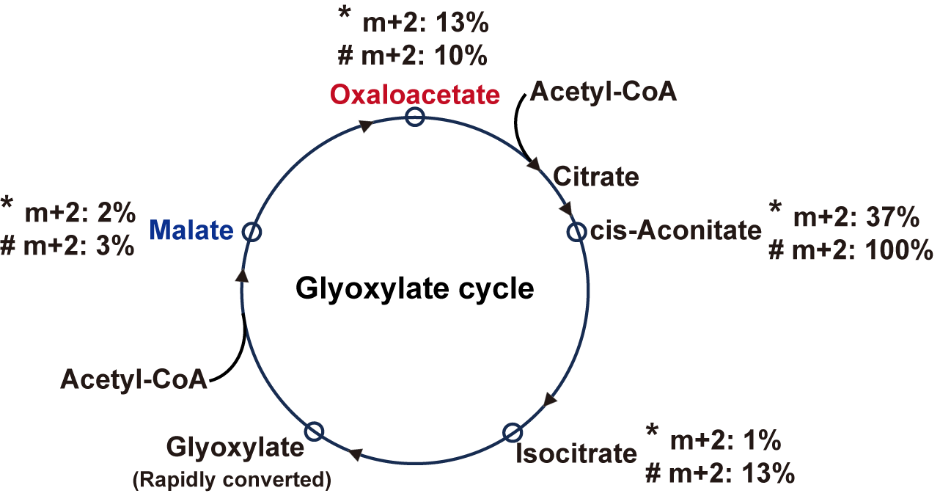


**Fig. S8 Key intermediates of the glyoxylate cycle were detected in the metabolic flux analysis.** Schematic diagram depicting the glyoxylate cycle. * indicates the U-¹³C-palmitic acid metabolic flux system, and # indicates the U-¹³C-glucose metabolic flux system.

**Table S1**

| **Reagent** | **Final concentration** | **Source** |
| --- | --- | --- |
| Peptone | 20 g/L | A100636, Sangon Biotech |
| Yeast extract | 10 g/L | LP0021, OXOID |
| L-cystein | 1g/L | A610133, Sangon Biotech |
| Ascorbic acid | 0.2 g/L | A800296, Macklin |
| Maltose | 5 g/L | A111184, Sangon Biotech |
| Methyl cellulose | 1 g/L | M112868, Aladdin |
| Yeast synthetic drop-out medium supplements | 1.3 g/L | Y1501, Merck |
| FBS | 10% | 16000-044, Gibico |
| Streptomycin | 100 mg/mL | A100382, Sangon Biotech |
| Penicillin | 100 U/mL | A613460, Sangon Biotech |
| Vancomycin | 50 mg/ mL | A600983, Sangon Biotech |
| Ciprofloxacin | 10 mg/ mL | A600310, Sangon Biotech |
| Gentamicin | 20 mg/ mL | A506614, Sangon Biotech |
| Amphotericin B | 0.5 mg/mL | 171375, Sangon Biotech |

**Table S2**

| **Gene ID** | **Potential Functions in Genome Annotation** |
| --- | --- |
| TMU_00021836 | Alpha-keto-acid decarboxylase (E1 subunit of KDHC) |
| TMU_00002640 | Dihydrolipoyl dehydrogenase (E3 subunit of KDHC) |
| TMU_00010542 | Dihydrolipoyl dehydrogenase (E3 subunit of KDHC) |
| TMU_00021610 | Serine palmitoyltransferase |
| TMU_00005703 | Serine palmitoyltransferase |
| TMU_00002997 | Malate dehydrogenase |
| TMU_00001190 | Malate dehydrogenase |
| TMU_00001129 | Malate dehydrogenase |
| TMU_00015102 | Malate dehydrogenase |
| TMU_00003080 | Malate dehydrogenase |
| TMU_00014894 | Malate dehydrogenase |
| TMU_00011948 | Malate dehydrogenase |
| TMU_00000982 | Malate dehydrogenase |
| TMU_00020781 | Malate dehydrogenase |
| TMU_00004617 | Malate dehydrogenase |
| TMU_00009296 | Malate dehydrogenase |
| TMU_00021968 | Acetyl-CoA carboxylase |

The genomic sequence of *T.mu* was sourced from the recently reported literature (PMID: 38366179), and reannotation was performed by Shanghai Majorbio Bio-Pharm Technology. The re-annotated genomic information is provided in Science Data Bank (https://www.scidb.cn/en/anonymous/WjdmYVFm).
